# Managing critically ill oncological patients in hospital: a survey across all ICUs in the UK

**DOI:** 10.1186/cc11020

**Published:** 2012-03-20

**Authors:** C Gore, T Wigmore

**Affiliations:** 1 Royal Marsden Hospital, London, UK

## Introduction

The survival rates for oncology patients admitted to the ICU have improved significantly. The prognostic influence of the pre-admission oncological and treatment history is being questioned, the most significant impact being related to acute physiological status. In this survey, we sought to evaluate the awareness of overall mortality rates in critically ill cancer patients among intensivists in the UK.

## Methods

We surveyed intensive care lead clinicians in December 2011 in order to establish: a profile of the hospital and ICU they work in; their estimate of overall ICU mortality for critically ill cancer patients; the value of six outcome indicators in predicting mortality in two subgroups of oncological candidates for ICU admission; and the local management of acutely deteriorating cancer patients potentially requiring ICU care.

## Results

**Figure 1:**
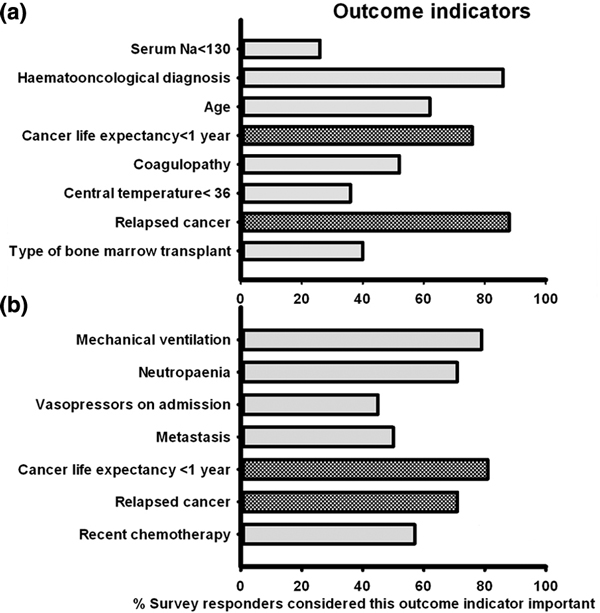
**(a) Hematological cancer**. **(b) **Solid tumor. Checked bars, not proven in the literature.

The ICU mortality rates estimated by survey respondents differed from those reported in the literature: for solid tumor 21% (SEM 3) versus 10 to 23%, for metastatic solid tumor 38% (SEM 4) versus 23%, hematological malignancy 45% (SEM 3) versus 33 to 43% with allograft transplant 54.8% (SEM 5) versus 39 to 50% and autograft transplant 56% (SEM 5) versus 44%. Regarding the management of cancer patients, there were conflicts reported between teams (rarely 44%, occasionally 56%, commonly 0.2%). Few units had established triage policies for the acutely ill cancer patient (5%) and it was also not common that plans were made prior to the patient's deterioration (never 11%, rarely 38%, occasionally 41%, commonly 9%). Figure 1 shows those outcome indicators thought to be important by responders in forecasting ICU prognosis.

## Conclusion

The awareness of improved outcome in critically ill cancer patients differs among physicians, and in general estimated mortalities were far higher than those reported in the literature.
